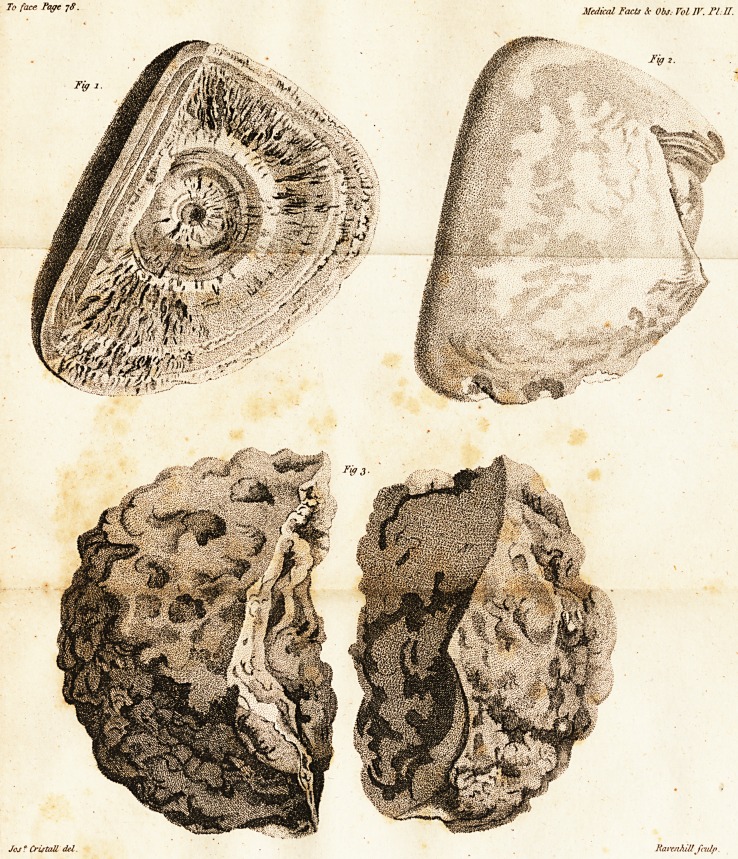# Observations on the Pathology and Mode of Treatment of Calculi in General, but More Particularly of Intestinal Calculi; with a Description and Chemical Analysis of the Intestinal Calculi of Horses

**Published:** 1793

**Authors:** William Gaitskell

**Affiliations:** Surgeon at Rotherhithe.


V. *Obſervations on the Pathology and Mode of Treatment of Calculi in general, but more particularly of Inteſtinal Calculi; with a Deſcription and Chemical Analyſis of the Inteſtinal Calculi of Horſes. By Mr.* William Gaitſkell, *Surgeon at Rotherhithe. Communicated in a Letter to Mr.* William Babington, *Apothecary to Guy's Hoſpital, and by him to Dr.* Simmons.

*To Mr.* Babington.

Dear Sir,

IT is with great diffidence I think of offering the following obſervations to the medical reader. Nothing but the importance and curioſity of the ſubject, the erroneous doctrines which have been entertained concerning it, and a deſire of promoting a more accurate inveſtigation of it, could ever have prevailed with me to publiſh them.

I am well aware that much more is neceſſary to complete the enquiry than will be found in the following obſervations; but as my frequent profeſſional avocations will not at preſent allow allow me to purſue the enquiry more minutely, I truſt this will plead my apology.

I have divided the paper into two ſections. In the firſt I treat of the pathology and mode of treatment of calculi in general, but more particularly of inteſtinal calculi; and in the ſecond I give a chemical analyſis of the inteſtinal calculi of horſes. In the latter of theſe ſections, I may, perhaps, by ſome be thought cenſurable, for having noted the materials of compoſition, without marking more exactly their proportions; but as different ſtones, chemically examined, differ ſomewhat in the quantity of their conſtituent principles, though not in their ſenſible qualities, I have been leſs anxious about critical minuteneſs.

My original deſign was to have made theſe obſervations the ſubject of a ſeparate publication, and to have requeſted leave to inſcribe it to you, as a ſmall, but ſincere, teſtimony of the reſpect and eſteem I have long entertained for you; but ſeveral reaſons, and particularly your friendly advice, have induced me rather to ſolicit a place for it in a work of eſtabliſhed reputation. I therefore beg leave to avail myſelf of your kind offer to tranſmit it to Doctor Simmons, to be inſerted, if he thinks proper, in the Medical Facts and Obſervations. In that that caſe I hope this letter will be allowed to appear by way of preface to the paper, that I may have an opportunity of expreſſing publickly the ſincerity with which I am, Dear Sir,

Your obliged Friend,
and humble Servant,

WILLIAM GAITSKELL.

*Rotherhithe,*

*May* 25, 1793.

### SECTION I.

### *Of the Pathology and Mode of Treatment of Calculi in general, but more particularly of Inteſtinal Calculi.*

NATURAL HISTORY informs us that calculi may be found in the ſtomachs and inteſtines of many quadrupeds; in ſome fiſhes, inſects, and worms; and likewiſe in the human body.

From the inteſtinal calculi I ſhall hereafter deſcribe, we may trace their affinity to the laminated ſtones named Bezoars by the ancients, and which ſeem to have been no other than theſe animal concretes*. To prove this more clearly, let us quote Dr.

* Bezoar ſtones were firſt noticed and employed medicinally by Avenzoar, an Arabian phyſician, who flouriſhed about

Dr. Lewis's defcription of them, as " preternatu-" ral or morbid concretions formed in the bodies " of land animals." Of thefe, he obferves, the oriental is of the fize of a kidney bean, of a roundifh or oblong rounded figure, of an even fmooth furface, and of a fhining olive or dark green colour; which, on being broken, appears compofed of a number of concentrical coats, of which the inner is fmooth and gloffy as the outer; in the middle, he adds, is either a cavity or fome powdery matter, or fome fmall bits of the leaves or ftalks of plants, or other like fubftances *.

This defcription feems correct, except in limiting thefe productions to land animals, for they are fometimes met with in fifhes. Therefore, to elucidate this fubject, I fhall enumerate the animals that bezoars are moft frequently found in. Among quadrupeds we may reckon about the eleventh century; but they were firft accurately defcribed by Garcias dal Horto, phyfician to the Portuguefe viceroy of the Indies. They took their name from the Perfian word *Badzcher*, which fignifies antidote, being confidered as remedies againft poifon. Even at this day they are faid to be in great efteem among the Perfians.

* Lewis's Materia Medica, 4to. 1768. p. 138, 139.

the the horſe, ox, goat, ſtag, the mountain deer of the Alps, the Braſilian monkey, and the porcupine; among fiſhes, the *phyſeter macrocephalus* Linnæi, or ſpermaceti whale; among inſects the *aſtacus fluviatilis,* or river crayfiſh; among cruſtaceous worms the *concha margaritifera,* or mother of pearl oyſter; and laſtly, mankind.

The Gentleman's Magazine, vol. vii, p. 448, gives an account of a ſtone taken out of the ſtomach of a horſe aged ſeventeen years, the greateſt circumference of which was 28 inches, and the leaſt 25. Its figure was an oblong ſpheroid; its weight 19 pounds avoirdupois.

In the 60th volume of the ſame work, at page 18, we have a deſcription, with a figure, of a cluſtered ſtone, weighing nineteen ounces; and an account of another as big as a penny loaf, like a heap of hardiſh horſe dung, from the inteſtines of a horſe thirty years of age. At page 885, of the ſame volume, four more inteſtinal ſtones are delineated, the firſt of which weighed one pound ten ounces; the ſecond, two pounds nine ounces; the third, eight ounces; and the fourth, ſeven pounds fourteen ounces. In the Philoſophical Tranſactions, vol. 48, we have an account of

 a mare, a mare, from whofe body a ftone was taken that weighed fifteen pounds twelve ounces. And Ruyfch preferved in his collection two calculi, which, with thirty-four others of different fizes, had been voided by a horfe in the Emperor's ftables at Vienna, in the fpace of fix weeks. The nucleus of one of thefe ftones was found to be a grain of barley*.

Mercatus has defcribed and delineated an oriental concretion of this fort in the Vatican collection †, prefented by the King of Portugal to Cardinal Alexandrinus, which weighed fomewhat more than four ounces; and alfo another from an animal of the ftag kind, brought to Rome by the Jefuits from Peru, the weight of which was fifty-fix ounces ‡.

In the monkey thefe concretions are found in the ftomach, of about the fize of an hazel

* Vide Frid. Ruyfchii Thefaur. Anatom. fecund. p. 39, 4to. Amftel. 1722.

† Mich. Mercati Metallotheca, (Armar. viii. de lapidibus animalibus innatis, cap. i.) folio, Romæ, 1719.

‡ Monardes, in his work, tranflated by Clufius, under the title of *Simplicium Medicamentorum ex novo Orbe delatorum Hiftoria*, has inferted a letter written to him by a correfpondent in Peru, who defcribes the pouch, communicating with the ftomach, in which thefe concretions are formed.

nut, harder than the other kinds, and of a dark greenifh colour, approaching to black. But in the porcupine, they are feated principally in the gall bladder, and are fimilar to biliary calculi in other animals.

In fifhes we may adduce ambergris, as a concretion of the fpermaceti whale. Dr. Swediar, in the feventy-third volume of the Philofophical Tranfactions, gives a fatisfactory hiftory of this animal product, and afferts, that it is found in the belly of the whale, and only of that particular fpecies called by Linnæus *phyfeter macrocephalus*. He defcribes it as being fituated about fix or feven feet from the anus, and never higher up; which, in all probability, fays the Doctor, is the inteftinum cæcum, hitherto erroneoufly confidered as a peculiar bag for the fecretion of this fingular fubftance. Intermixed with this are a number of black fpots, apparently the beaks of the *fepia octopodia*, which is the natural food of this fpecies of whale. All whales, it feems, which afford this concretion, are found fickly and emaciated; but healthy whales never produce any *: therefore we may conclude it to be an animal concrete, generated

* See Phil. Tranf. vol. 81, page 43.

by difeafe, and which proves fatal by its mechanical ftimulus *.

In the infect we confider oculi cancrorum to be the ftony concretion of the cray fifh: they are thus defcribed by Doctor Lewis †:—" about " the fize of peafe, of a roundifh fhape, flat- " ted on one fide; in colour white; fome- " times with a reddifh, and fometimes with a " blueifh caft;—internally of a leafy texture‡."

In the worm may be inftanced the pearly concretions of certain oyfters—the *concha margaritifera*. Thefe concretions are of a bright femi-tranfparent whitenefs, and are of two forts,

* Clufius was of opinion, that ambergris was the indigeftible part of the food collected in the ftomach of the whale; and Kempfer fpeaks of it as excrement, and mentions, that the Japanefe, for this reafon, call it *kufura no fuu*, i.e. whales dung. But, I think, from all whales being found unhealthy in which it is difcovered, and the quantity imported being fmall compared with the quantity that would every where be to be found were it natural fæces, in thofe feas where the fpermaceti whale inhabits, we may fafely conclude that it is a difeafed product, fimilar to ftones in the inteftines of horfes, and of other animals.

† Materia Medica, p. 172.

‡ Geoffroy afferts that cray-fifh change their ftomachs and inteftines at certain periods; that when the ftomach is renewed the old one becomes fubject to the digeftive procefs: and that after this procefs concretions are found in the new ones. —Suite de la Mat. Med. de M. Geoffroy, tome i, p. 338.

the the oriental and occidental. The oriental are of a ſilver hue; the occidental of an opaque white: and they are found on the inſide of the ſhell *.

In the human body we have too many well recorded facts of inteſtinal concretions, and the direful effects they are productive of; ſome of which ſhall now be ſelected.

Lanzoni mentions † the caſe of a woman, in whoſe ſtomach were found ten ſtones, the largeſt weighing an ounce.

Dr. Coe, in his treatiſe on biliary concretions, gives an inſtance of a woman, from whoſe rectum was extracted a concretion, the nucleus of which was a plumb ſtone ‡. In the Edinburgh Medical Eſſays §, we have an account

* As neither crabs eyes nor pearls are found in all cray fiſh or all oyſters, and there is no ſuggeſting any uſe that they can furniſh to the œconomy of theſe animals, we may, perhaps, be juſtified in concluding that they are formed by diſeaſe in their inteſtines. The poſitive confirmation of this hypotheſis may be difficult, but it may in time perhaps be decided, by the obſervation of diligent naturaliſts. See Reaumur's paper on the formation of pearls and ſhells, in the Memoirs of the Academy of Sciences at Paris, for the year 1717.

† Acta Phyſ. Med. Nat. Curioſ. vol. i, p. 117.

‡ Coe on Biliary Concretions, p. 137.

§ Vol. 1. p. 243.

of a ſimilar fact. In this laſt caſe the ball was of an irregular cubical ſhape, and weighed five drachms. In the Eſſays and Obſervations, Phyſical and Literary, is the hiſtory of a boy who had three ſtones extracted from the rectum, the nuclei of which were the ſmall bones of ſheep's trotters *.

The third volume † of the Memoirs of the Royal Academy of Surgery at Paris, contains an account of a woman from whoſe bowels was taken a ſtone weighing two ounces two drachms and a half.

In the Philoſophical Tranſactions, vol. 27, we have an account of a concretion formed upon a plumb ſtone, and retained in the inteſtinum cœcum; and in the London Medical Journal, Vol. 6, p. 355, Mr. Johnſon, of Lancaſter, relates the caſe of a woman who voided, by ſtool, a ball of hardened fæces, weighing three quarters of an ounce, the nucleus of which was alſo a plumb ſtone.

Theſe facts, (and other ſimilar ones might eaſily be collected from books) are ſufficient to ſhow that inteſtinal calculi are not limited to

* Vol. 11. p. 345. † P. 56.

the quadruped; and that they occur more frequently in the human body than has perhaps been generally ſuſpected.

I ſhall now offer ſome obſervations on the formation and growth of animal ſtones, and on the ſtate of the organs which produce them.

When my mind was firſt occupied in the inveſtigation of this ſubject, it was forcibly impreſſed with a ſeeming conviction of the truth of the prevailing doctrines, that ſtone was a calcareous depoſition from urine, or calcareous earth ſecreted with mucus, which for the formation of ſtone required only a proper nucleus to attract it*. But when I had peruſed the work

* It has been ſuppoſed by the ancients, and by the moſt eminent among the moderns, that urine contains the elements of ſtone, is more or leſs ſaturated with calcareous earth, and prepared to accumulate on any inſoluble body. The juſtly celebrated Dr. Black, Profeſſor of Chemiſtry at Edinburgh, delivers this doctrine. Dr. Monro, Profeſſor of Anatomy there, contends for the ſame; and Dr. Boerhaave, with his learned commentator, Van Swieten, have been of a ſimilar opinion. But when ſtones have been diſcovered where no urine exiſted, their origin has been aſcribed to the ſecrework lately publiſhed by Doctor Auſtin on this ſubject, and had repeated ſome of his experiments, I became convinced of the truth of his opinion: therefore, in viewing the origin of ſtone, I ſhall adopt the ſentiments of this ingenious writer, and attempt to prove, that concretions in animals are morbid in their origin, that the urine contributes nothing to their formation, that they are compoſed of a modified mucus, and are not calcareous.

We can hardly believe any organ or organs of an animal body to perform their natural functions, when they become the agents of productions injurious to the ſyſtem; therefore, if glands or glandular membranes ſhould ſo alter the quality of their ſecreting liquors, as to produce an hurtful inſtead of uſeful fluid, we properly nominate it diſeaſe. When the ſalivary glands, under hydrophobic irritation, prepare a poiſonous fluid in place of mild ſaliva;

ſecretion of calcareous matter in combination with mucus, which cryſtallizing on the ſurface of ſome nucleus, formed that incruſtation called ſtone. However, we ſhall ſee that theſe opinions are ill founded, raiſed upon external appearances, and not ſupported by experiment.

or or the glands of the breaſt a cancerous fluid in place of milk; or the follicles of the urethra an opake venereal mucus in place of what is innoxious; theſe are unnatural actions, and morbid products; which, as each differs ſpecifically from the others, aſſumes ſome diſtinguiſhing character. Upon the ſame principle, that affection of follicular membranes which ſecretes mucus with the property of concretion, may be characteriſed with the epithet *lithic*. The impoſſibility of deſcribing the peculiar mode of action which the living ſolids take on, or their peculiar mode of operation in changing the chemical and ſenſible qualities of their contained juices, is no objection to this reaſoning, while the ſudden alteration in the quality of ſecreted liquors by the application of various irritants, gives ſuch ſtrong confirmation of it. From theſe pathological data we conclude the following to be the conſtruction of ſtone: A mucus is depoſited with the property of coagulating, which invelopes the body that irritated the veſſel to produce it—this, when conſolidated, becomes the nucleus to a new covering, which conſolidating like the former, receives an additional coat; to this is added a fourth, and ſo on on *gradatim*, till numerous coats are given to it*. That ſtones are thus generated, and in this way

* This intermitted depoſition of coagulating mucus, ſhows, that the veſſels ſupport their actions for a determined time, and then become ſuſpended. After ſome ſuſpenſion or diminution of action, the proceſs is reſtored again; without this, no increaſe of magnitude could ariſe, or regular laminæ be formed. Sometimes the actions deſtroy themſelves without renewal, in which caſe the ſtone is ſmall, and not coated; and ſometimes the actions, after temporary ſubſiſtence, are deſtroyed by the application of new ſtimulants. With reſpect to the ſubduction of lithic action by new ſtimulants, let us quote the words of Dr. Auſtin: "The "removal of a ſtone by the operation of lithotomy, often "proves an effectual and permanent cure. It would not be "eaſy to account for this, if the origin of the ſtone be re-"ferred to the kidnies; and if it be referred to the general "ſtate of the habit, ſuch an effect would be abſolutely un-"intelligible. But if the bladder, with the glands and "membranes contiguous to it, be the ſeat of this diſeaſe, "the change induced in theſe parts by lithotomy, will be "ſufficient to account for the cure. For in this operation, "the neck of the bladder, the proſtate gland, and the "lower part of the urethra, are divided; the courſe of "the urine is changed; the ſtone is extracted; and a large "communication made with the open air: thus a long-"continued irritation is removed, and a new ſtimulus ap-"plied to theſe parts. The effects of this are not more "evident in the ſtone than in other diſeaſes of the urinary "bladder." Auſtin on Stone, p. 76.

augmented, augmented, appears from their concentrated ſtructure; all ſtones, of whatever ſize, when ſawed through the centre, diſplaying an arrangement of circles, and this with very few exceptions.

That concretions can be formed without the aid of urine, we have ſhown from a variety of facts. The urine can exert no influence in cavities where it has no acceſs, therefore it can contribute nothing to the formation of ſtone in the cavities of the inteſtines, gall bladder, bronchial veſicles, ſalivary ducts, or follicles of the ſkin; yet, all theſe, and many more, have been the ſeats of ſtone, not differing from thoſe diſcovered in the urinary organs.

Let us now examine another opinion, whether there be an acid *ſui generis*, ſecreted from the blood, called the lithic acid of Scheele and Bergman, with the property of coagulating the animal matter it combines with?

Mr. Scheele aſſerts, that all calculi taken from the bladder or kidneys, have this acid in their compoſition *, the remainder being ſalts of the urine and animal earth; but Dr. Auſtin, in his

* The nature of the lithic acid of Scheele will be more particularly noticed in the chemical analyſis of ſtone.

accurate accurate analyſis of calculi, was unable to trace this acid; even the urinous cruſts of walls, ſelected for the purpoſe, and ſublimed in a retort, were not able to afford it him: therefore, he concludes, that it only appears in ſome calculi, is deficient in others, and not neceſſary to the formation of any.

An anonymous but ingenious author, in the year 1787, publiſhed a work in confirmation of the univerſality of this acid, entitled, "A Treatiſe upon Gout and upon Gravel, in "which the Sources of each are inveſtigated," and he attempts to prove, that the lithic acid is always preſent in the ſyſtem; that it is in combination with animal earth; and can be precipitated from this earth in a concrete ſtate, by the introduction of any other acids, whether mineral, vegetable, or aërial. This writer goes farther, and aſſerts, that the animal earth is ſometimes ſuperſaturated with it, in which caſe the acid will be precipitated ſpontaneouſly; that conveyed through the kidnies it forms gravel in the bladder; or if detained in the blood-veſſels, gout. The following are his words: "A precipitation of the concreting acid in the "fluids is attended with prejudice to other "parts of the ſyſtem, as well as to the urinary

" passages. As soon as the acid has been de-

" tached from the substance with which it was

" combined, it becomes a species of matter to

" the action of which the body is unaccus-

" tomed; and when the redundancy is very

" considerable, a deposition of the particles

" will take place in the blood-vessels, so as to

" give an interruption to the freedom of cir-

" culation. In consequence of this interrup-

" tion, there frequently arises a peculiar affec-

" tion of the inflammatory kind, and that af-

" fection is gout *."

If this hypothesis were just, I should suspect, that in tropical climates, where acids are freely employed, gout and stone would be frequent occurrences; but experience shows us quite the reverse; for in those climates where the natives subsist principally upon vegetables and fruits, these diseases are rare to be met with †.

More-

* See pages 63 and 64 of the work here referred to.

† Dr. Moseley mentions the stone in warm climates as a very uncommon disease, and relates the remarkable circumstance of the benefit of changing the climate in this affection. It is the case of an officer of the 79th regiment, who had been greatly afflicted with the stone in England; but by going to Jamaica, and residing there three years, the disease gradually

Moreover, in northern climates, where both these diseases are prevalent, acids are most sparingly employed; while animal food, which is powerfully alkalescent, is liberally used, without proving an antidote to either. Besides, many people have been martyrs to arthritic affections without any concretions being formed in their bodies; and when they be formed, it is not experimentally proved, that the acid of Scheele makes part of this compound *.

I have gradually diminished, and entirely left him. The stone was so large, and the disease so violent in England, that, on examination, Mr. Pott recommended the extracting it by lithotomy, to which the officer consented, but which operation was fortunately avoided by his being suddenly obliged to join his regiment.

* Mr. Watson, in his paper on the dissection of a gentleman who died of the gout, draws the following conclusions on arthritis, and its concretions:

" Is it not remarkable," says Mr. Watson, " where we " had so much of the distemper, that there should have " been no marks of it in any of the hollow viscera; neither " in the kidnies, liver, spleen, or pancreas?

" It has been, I believe, a pretty common opinion, that " those who have gouty concretions in their joints, are very " liable to the stone in the bladder and kidnies; as if the " one disease were generally productive of the other.

" Is not this pronouncing rather too much? For of all " the patients cut in our hospitals, men, women, and chil-

" dren,

I have repeated the author's experiments of precipitating fome cryftalline particles from urine (called the lithic or concreting acid) by means of the muriatic acid, and have uniformly fucceeded. The vitriolic and nitrous acids produce the fame precipitation, with fome little difference in colour; that by the muriatic being orange coloured, while the other two are of a

" dren, how few do we meet with that have even the flighteft " indication of gout about them?

" Both the gout and the ftone are morbid fecretions, and " may poffibly exift together in one and the fame fubject; " but differ effentially in their material principles, and " have very different tendencies. The calculous matter is " formed in the urinary paffages—the gouty depofits itfelf " generally on bones, cartilages, membranes, and lymphatic " glands. The gouty feems to be a kind of earth, different " from that which generally forms a ftone in the urinary " bladder, for it never appears lamellated, or to have any " kind of nucleus, but is white, foft, and uniform through- " out: it may be diffolved, and being ground down by the " motion of a joint, readily mixes with the fynovia, form- " ing a fmooth creamy fluid.

" The gouty earth is then a kind of greafy bole, which " may eafily be made to mix with oil and water, which in " general the calculous cannot be made to do; fo that in " every refpect, in colour, form, and confiftence, it feems " to differ effentially from that which lays the foundation, " and caufes the increafe of the ftone in the bladder."— Med. Communications, vol. 1. art. 3.

 pale pale yellow. With the acetous acid I have alſo ſucceeded.

The moſt healthy urine contains this particular powder, or concreting acid, as it is called, in ſolution, which may be precipitated in the quantity of about half a grain from ſix ounces of urine, in the ſpace of twenty-four hours, by thirty drops of either of the concentrated mineral acids. My ſolution of this powder in the nitrous acid has not furniſhed the phænomena deſcribed by Scheele as peculiarly characteriſtic of lithic acid, viz. a deliqueſcent red maſs, communicating its colour to linen: therefore my concluſion is, that whether it be lithic acid, or ſome other acid united with animal mucilage, it may contribute a part to the compound of concretions in the urinary bladder, but ſeems no way neceſſary to the act of concretion, as theſe ſubſtances can be perfected without it, and are frequently ſeated where the urine can have no influence in their production.

When foreign bodies obtain admiſſion into the bladder, as bougies, catheters, &c. an incruſtation is obſerved to take place; but this incruſtation is not lithic cryſtals, but follicular mucus. The follicles become ſtimulated to more copious ſecretion. This ſecretion is not natural natural mucus, but mucus changed in its properties *. The change takes place where the ſtimulus is applied, and becomes limited or extended, according to the diatheſis of the part, and morbid irritability of the bladder. Sometimes the whole internal membrane of this muſcular cavity is covered with a ſtony cruſt, and ſometimes only a ſmall portion of it partakes of the affection.

I have attempted the formation of artificial calculi, by ſuſpending ſomething inſoluble in urine; but have never been able to effect it.

* Van Helmont was of opinion, that a coagulating principle was derived from the urine, and was peculiar to the human urine alone; this coagulating principle, however, is not reſident in the urine, but in combination with mucus.

We need not be ſurpriſed at the veſſels of animals ſecreting fluids with the property of coagulating, when we ſee vegetables, as the *arundo bambos* of Linnæus, produce concretions ſimilar to flint. It has been lately proved by chemical analyſis, that the Tabaſher, found in the joints of the female bamboo, and ſo accurately deſcribed by Dr. Ruſſel in the 80th volume of the Philoſophical Tranſactions, is truly ſiliceous. And in Dr. Pitcairn's hot-houſe at Iſlington, there was lately a bamboo, containing a ſiliceous pebble in the joint of it, formed by the juice ſecreted from its veſſels.—See Phil. Tranſ. vol. 80, 81.

A piece of ſlate pencil, appended to a ſtring, was ſteeped in a phial of urine; the urine, to prevent decompoſition by putrefaction, as well as to correſpond more exactly with what ought to take place in the bladder, was renewed three times a day for a month; at the expiration of which no incruſtation had taken place. This being repeated, and the time of ſuſpenſion extended, was followed with ſimilar conſequences: therefore I conclude, that the following aſſertion of Baron Van Swieten is probably conjecture without proof: "If the ſmalleſt quill be dipped in "healthy urine freſh made, it acquires a cruſt "of very ſoft ſand, which adheres to it; and "when freſh urine is again poured on it, "increaſes in quantity. Thus the ſtone may "be generated out of the body, namely, when "another ſolid body is put into the urine; "to which, as to a baſis, the elements of "the ſtone are collected, and adhere. Whence "the material cauſe of the ſtone exiſts even in "the moſt wholeſome and ſound fluids of the "body, but an indiſſoluble ſubſtance, exiſting "in ſome particular place in the body, affords "the occaſional cauſe, attracting to itſelf the "elements of the ſtone, even in perſons in

"whom

"whom no propenſity to the ſtone was ever "obſerved *."

But what the urine of a calculous patient may contribute to the augmentation of ſtone in the bladder, I have never had an opportunity to experience—that ſomething may be added, we have the authority of Doctor Auſtin for: he took two ſtones of equal ſize—one was placed in mucus from the bladder of a calculous patient, the other in urine decanted from this mucus, and ten times as much in quantity. In the latter it received ſome augmentation—in the former conſiderably more; and in every reſpect ſimilar to what the patient had diſcharged from his bladder †.

Having now adduced many facts in ſupport of my opinion, that concretions may be formed in animals without the aid of urine, that they are morbid in their origin, and compoſed of a coagulating mucus, I ſhall proceed, in the next place to conſider what may be the ſymptoms they are fitted to produce, and the remedies adapted to relieve them.

* Van Swieten's Comment. vol. 16 of the Engliſh Tranſl. 8vo. Lond. p. 113.

† Auſtin on the Stone, p. 9.

Were this ſubject to be diſcuſſed fully, it would lead to a wide field of enquiry; I ſhall therefore confine myſelf to the following points of view—the preſence of ſtones in the alimentary canal, their effects on the ſyſtem, and the particular modes of relief in ſuch caſes. My pathology of the origin of ſtones has been more extenſive than this, from an hypotheſis of its being one ſpecies of action, and one ſpecies of product; but my pathology of ſymptoms will be more limited, as every different ſituation has different ſymptoms, and requires ſome variation of treatment.

As my ſpecimens were extracted from horſes, my intention was, to have treated of the ſymptoms produced in this ſpecies of animal alone; but want of information excludes this attempt, and obliges me to conſider them in man, and apply them analogically to the quadruped. This application will probably not be conſidered as improper, when we reflect, that many diſeaſes in the horſe quadruped have the ſame ſource, progreſs, and termination, as in the human body; among which may be be enumerated, that which I propofe to defcribe, the ftone colic, or *colica calculofa* of Doctor Cullen. It is thus defined by that late fenfible and ingenious nofologift, " dolor abdominis, " præcipue circa umbilicum torquens; vomi- " tus; alvus adftricta; cum duritie in quadam " parte abdominis fixa; calculis quondam " per anum dejectis." Syn. Nofol. Meth. Gen. 59, Spec. 7.

That this definition is accurate and comprehenfive, the following cafes will illuftrate.

Doctor Monro, profeffor of anatomy at Edinburgh, was confulted in the cafe of a gentleman, who had a hard tumour under the ribs on the left fide. It had been taken for a difeafed fpleen; was attended with pain, and with many grievous fymptoms. The Doctor afferted the difeafe to be an inteftinal ftone, for which he ordered the patient oily purgatives, linfeed tea, folutions of foap, and change of pofture. The ftone removed, and got into the zygmoid flexure of the colon, where it was detained, and proved fatal. Here was the " durities in quadam parte " abdominis fixa."

Doctor Coe relates the cafe of a " woman, " who was thought to be afflicted with a ner- " vous colick, and made ufe of medicines

" proper for that diforder, from which fhe
" reaped fome benefit. The fymptoms after-
" wards changing, it was judged that a ftone
" was paffing from the kidnies to the bladder,
" which diagnofis feemed to be confirmed by
" this circumftance, that after a few days fhe
" found relief, but alfo felt a weight about the
" lower part of her belly. After fome inter-
" val of time, fhe was feized with a fudden
" violent inclination to go to ftool, and the fen-
" fation of weight increafed very much; at the
" fame time fhe felt a violent pain in the mid-
" dle of the gut rectum; prodigious, but ufe-
" lefs efforts to go to ftool, next followed, at-
" tended with cold fweats and faintings. She
" felt an hard body in the gut rectum, which
" catching hold of, and at the fame time ftrain-
" ing with all her force, it at length came
" away with the ftool, and being examined
" by a phyfician prefent, was found to be a
" ftone of the inteftinal kind, containing in its
" centre, for a nucleus, a fmall biliary ftone,
" round which the other calculous matter had
" grown *."

* Coe on biliary concretions, p. 37.

The ſubject of the caſe I have already referred to in the Edinburgh Medical Eſſays, vol. i. p. 243, and which I am induced to quote here more at large, was a girl, twelve years of age, who had been afflicted for ſix years with moſt acute pains in her belly, which returned by fits with extreme violence, and were increaſed from the uſe of acids, or any thing that was ſour, or hard of digeſtion. Theſe pains uſed to abate if the body were naturally open, or made ſo by means of laxative medicines, or clyſters. At length ſhe was ſeized with a more excruciating fit of her diſorder than ſhe had hitherto experienced, attended with violent retchings and an obſtinate coſtiveneſs. Dr. Simſon, who relates the caſe, being called in, tried many things without ſucceſs, ſo that for almoſt three weeks together the patient was in perpetual torment, and from pain and want of ſleep and nouriſhment, became totally emaciated; though before a healthy freſh-complexioned girl. At length, when a cure of ſo obſtinate a diſeaſe was almoſt deſpaired of, ſhe began to vomit bile of a deep yellowiſh colour; ſhe was adviſed to drink plentifully of warm water, and frequently; which ſhe readily did. After having vomited ſix or ſeven times, ſhe had a plentiful ſtool, and felt an an hard ſubſtance paſs through the anus, which, upon examination, proved to be a ſtone of an irregular cubical figure, four inches in circumference, and which weighed five drachms. In the centre was a plumb ſtone, to which the calculous matter had concreted in layers.

This caſe, and the preceding one from Coe, comprehend both the genus *colica*, and its particular ſpecies *calculoſa*. But the ſpecies is not completely defined, as there is a want of the "durities in quadam parte abdominis fixa," yet it is very well characterized, by the "calculis per anum dejectis." Therefore we have the ſpecific diſtinction of colic as complete as the nature of the caſe will admit; for in no one inſtance are the characters of diſeaſe ſo fully united, as to diſplay themſelves in every patient *.

Other animals become great ſufferers from the mechanical irritation of calculous concretions

* Dr. Cullen has reduced the number of his ſpecies of colic as much as poſſible, and in this he ſeems to be right; but when he aſſerts that his ſeventh ſpecies, or colica calculoſa, admits of no difference of treatment from the calloſa and ſtercorea, in this I beg leave to diſſent from him, becauſe the ſpecies in queſtion requires all the treatment competent to the other two, and ſome treatment peculiar to itſelf.

in in the inteſtines, particularly when of large ſize, and the animal incapable of diſcharging them. Thus whales, whoſe inteſtines contain ambergris, are always found ſickly and emaciated *; and horſes, whoſe inteſtines contain large ſtones, are often found to die of enteritis †. As mechanical irritation, long continued, may deſtroy life, without previous inflammation, and as this ſeems to happen frequently to the whale ‡, ſometimes to the horſe, and alſo to the human ſubject, the following intereſting narrative of its effects upon a ſhark, is ſelected by way of illuſtration.—" Some fiſhermen, fiſhing in the " river Thames, near Poplar, with much dif-" ficulty drew into their boat a ſhark, yet " alive, but apparently very ſickly; it was taken " aſhore, and being opened, in its belly were " found a ſiver watch, a metal chain, and a " cornelian ſeal, together with ſeveral ſmall

* See Philoſophical Tranſactions, vol. 81, where there is an account of 362 ounces of ambergris, taken out of the bowels of a female ſpermaceti whale; the animal was old, and much emaciated.

† Gent. Magazine, vol. 60, p. 18, 895.

‡ The phyſeter macrocephalus Linnæi, is the only ſpecies of whale in which ambergris is found, and which is therefore the ſubject of this diſeaſe.

" pieces

"pieces of gold lace, ſuppoſed to have be-

"longed to ſome young gentleman, who was

"unfortunate enough to fall overboard, but

"that the other parts had either been digeſted,

"or otherwiſe voided, but the watch and gold

"lace not being able to paſs through it, the

"fiſh had thereby become ſickly, and would

"in all probability very ſoon have died *."

The ſtomach and inteſtines of moſt animals ſeem endowed with ſuch exquiſite ſenſibility, and ſuch general influence on all the other functions of the ſyſtem, that we need not wonder at theſe miſchiefs—and the caſes we have ſelected ſhow, that the human ſubject, horſe quadruped, and a certain ſpecies of whale fiſh, are the moſt frequent objects of it; therefore it behoves the medical practitioner to diſcriminate the preſence of inteſtinal calculi in the human body, while the veterinary practitioner diſcriminates their preſence in the quadruped.

Horſes are known to be ſometimes attacked with ſevere colicky affections; and if, during a paroxyſm, ſome concretions ſhould be evacuated with their excrement, the diſeaſe be-

* Dodſley's Annual Regiſter, chronicle part, page 227, year 1787.

comes comes evident, and juſtifies the idea of unevacuated concretions being the cauſe of it. When ſmall, they are ſeldom productive of uneaſineſs, being for the moſt part rejected with the fæces; but when large, collected in numbers, detained in the ſtomach, inteſtinum cœcum, or zygmoid flexure of the colon, the parts become incapable of relief, and art muſt be called in to afford it.

In contributing this relief, let us take nature for our guide, and purſue the path ſhe ſo wiſely points out. We perceive the ſtomach and inteſtines of the human ſubject rouſed by nature to evacuate indigeſtible ſubſtances, as ſoon as they become hurtful by their preſence; therefore, when the huſks of grapes, plumbs, currants, the ſtones of any of theſe fruits, or marbles, dumps, pieces of money, or other inſoluble bodies, are received into theſe cavities, they ſoon become ſtimulated to reject them—this induces us, when the powers of nature prove inadequate, to employ remedies adapted to accompliſh it; and theſe remedies are purgatives: therefore, by analogy we infer, the ſame applications to be proper in the colica calculoſa of horſes. With the adminiſtration of purgatives to horſes in this diſeaſe, it will be advifable to combine diluents of the mucilaginous or oily kind, both given by the mouth, and injected by the anus.—Befides, it will be prudent, if tenfion of the abdomen, and unabating pain be prefent, to let copious bleeding be premifed. The diluents will be of great utility, both in a mechanical and chemical view—for by their bulk they will tend to propel the concretions toward the anus, and by their chemical operation on the mucus which always furrounds thefe concretions, they will loofen their attachment to the inteftine, and in this way facilitate their expulfion*. The purgatives to be made choice of, may be foap and aloes, calomel and jalap, or glauber's falt largely diluted, interpofing an opiate to diminifh the conftrictions, if inflammation fhould not prohibit it.

Should thefe remedies fail in their effect, and no fymptoms of actual inflammation be prefent,

* Diluents fhould always be largely employed.—They fhould be thrown in to the quantity of fome gallons by the anus, and an infufion of oatmeal, or bran tea, will be very fuitable for this purpofe. To thefe may be added fome culinary falt, or a folution of foap, to promote the periftaltic motion of the inteftines.

but the animal continue to languifh *, two other modes may be adopted—either the extirpation of the ftone by mechanical means, or its folution in the body by folvents.

The mechanical means may be fourfold—dilution, excifion, the hand, and forceps. The firft has been already fpoken of; the fecond muft be extremely hazardous, unlefs performed on the verge of the anus; the third and fourth can only be practicable when the ftone gets into the rectum, and then they may be employed with advantage. Hardened horfe dung in the dry belly ache has often been extracted by the hand of the farrier, when the gut was incapable of expelling it; the fame has been effected in the human body: I have given affiftance with the hand and fcoop, when the patient has been in the greateft diftrefs, and the gut fo weakened by diftention, that the moft violent ftrainings were

* Van Swieten mentions that whole oats are fometimes the nuclei of concretions in horfes, and afferts, as a well-known fact, that grooms mix ftraw cut fmall with the oats which they give their horfes, that thofe animals may be made to chew the oats, which otherwife they would fwallow whole: without this precaution, thefe animals are frequently obferved to languifh and decay. See his Commentaries, Sect. 1414.

unable unable to procure relief. Clyſters were in this caſe ineffectual, being inſtantly returned upon injection; however, in every inſtance, they merit trial and repetition; for, if hardened excrement be the cauſe, they may ſoften and divide it; if calculus, they may ſtimulate the inteſtine to expel it.

Previouſly to the introduction of the hand, warm oil ſhould be thrown up, and the hand well lubricated with the ſame; this will allay irritation, and prepare the parts for the eaſy expulſion of the ſubſtance.

The caſe related in the Eſſays and Obſervations, phyſical and literary, and which I have likewiſe referred to in a former page of this paper, was that of a boy who, " after eating " ſheep's and lamb's trotters greedily, and " ſwallowing ſome of the ſmall bones, lan- " guiſhed in a ſhocking manner for ſix years, " being afflicted during the whole of that time " with frequent and violent pains in his bowels, " till at length three ſtony balls being ex- " tracted from the anus with a pair of forceps, " and two others being voided by ſtool, he got " rid of all his complaints, and in a ſhort time " recovered his health and ſtrength. Upon

" breaking

" breaking two of these stones, a small flat " bone was found to be the nucleus *."

When the stone exhibits no visible mark of its presence, either by external protuberance, detention in the rectum, or expulsion of small ones, but yet, from the frequent returns of colic, constipation of the bowels, and abdominal distention, irregular fever, and daily emaciation, there is reason to suspect its existence; after trying the power of purgatives, we should aim at its solution by suitable remedies. †.

The action of solvents is two fold; they either act on the surface of the stone directly as menstrua, causing it to waste, and be evacuated, in every cavity where they have access; or they act on the membranes which generate it, diminishing the lithic irritation, and sometimes totally destroying it. In fact, if this class of

* In the third volume of the Memoirs of the Academy of Surgery at Paris, p. 56, is an account of a stone weighing two ounces two drachms and a half, which being too large to be extracted by the forceps, required several incisions previously to be made in the anus.

† This attempt at solution points out the necessity of distinguishing the calculous colic, as it harasses for years before it destroys, and may chance to be relieved by suitable menstrua.

remedies can prove useful, and in some instances they have shewn themselves so, after passing the whole round of circulation, previously to the exertion of their powers, how much more may we expect from them, when employed with concentrated influence, on calculi seated in the intestines?

These antilithics and solvents are caustic fixed alkali, caustic mineral alkali, lime water, soap, beards of leeks, and uva ursi. The four first, are properly solvents, though they sometimes act on the membranes. The two last, are more properly antilithics, as they act primarily on the membranes, while their chemical action is unknown.

Before we employ any of the alkalies as solvents, it will be right to adopt the rule of Mr. Lane, who recommends, if any stones be evacuated, to try the power of chemical menstrua, prior to the internal exhibition of them; for this gentleman found, that different stones were differently acted on; and that different laminæ of the same stone were differently affected by the same agent*.

* See the experiments of Mr. Lane in the Philosophical Transactions, Vol. LXXXI, and in the Medical Facts and Observations, Vol. III.

Doctor

Doctor Auſtin obſerves *, that " if we con-

" ſult authors on the effects of alkaline ſub-

" ſtances and lime water in this diſeaſe, we

" ſhall find, that they ſometimes afford relief

" to the painful ſymptoms of the ſtone, without

" effecting a ſolution of it; that ſometimes

" they radically cure the diſorder, by diſſolving

" the ſtone, and removing the diſpoſition to

" generate ſtone; and that at other times they

" are attended with no beneficial effects."

The ſame author quotes from Dr. Whytt, the facts reſpecting the ſolvent powers of lime water and ſoap, in the caſe of the Right Rev. Dr. Newcome, Lord Biſhop of Landaff. This prelate, while drinking two Engliſh quarts of lime water daily, for the cure of the ſtone in the bladder, poured his urine every morning and evening upon a piece of human calculus, weighing thirty-one grains; by which in the ſpace of four months it was reduced to three pieces, weighing in all only ſix grains. Upon one of theſe pieces, weighing 2. 31 grains, he cauſed to be daily poured, for two months, the freſh urine of a perſon who drank no lime water; at the end of which time, the piece was

* Auſtin on the Stone, p. 85, 90.

found to weigh 2. 56 grains, having increafed in weight a quarter of a grain. This fame piece being afterward fteeped in the Bifhop's urine (who continued to drink lime water as above) from June 24th to July 9th, was in thofe few days quite crumbled into powder *.

"Since this experiment," obferves Dr. Whytt, "fhews, beyond difpute, that lime water, "unaffifted by foap, can communicate to the "urine a power of diffolving the ftone out of "the body, it can fcarcely be doubted, that it "muft have the like effect on it when lodged "in the bladder. And that the diffolution of "the ftone in the bladder has been completed "by foap alone, appeared evidently in the cafe "of the Rev. Mr. Matthew Simfon, Minifter "of Pancaitland, near Edinburgh; an account "of which will foon be made public by Doctor "Auftin, who opened his body after death. "Mr. Simfon had, from 1730, been afflicted "in a lefs or greater degree with the fymptoms

* The perfon who tried the folvent powers of his urine upon this calculus, without drinking lime water, fhould have taken the fame bulk of pure foft water, and then marked its effects; probably the folution might have been equally well effected as by the Bifhop's urine, who had drank the lime water, as pure water fhows fome degree of action on calculus.

" of a ſtone in the bladder, and in November

" 1735, was ſounded by Doctor Drummond

" of Perth, and Mr. Balderſton, Surgeon, in

" Edinburgh; by whom a ſtone was not only

" plainly felt, but alſo by the patient himſelf.

" In February 1737 he began to take ſoap, and

" after 1743, never had any graveliſh ſymp-

" toms. He died in May 1756, and when his

" bladder was looked into, there was neither

" ſtone nor gravel found in it*."

Now ſuch facts as theſe concerning the power of lithic menſtrua, upon ſtones moſt incommodiouſly ſituated for their action, ſhould flatter us with the moſt pleaſing expectations, and induce us to preſcribe them in caſes where their influence may be more powerful, as in the colica calculoſa of horſes.

In the exhibition of theſe ſolvents to horſes, the cauſtic vegetable alkali, or lime water, may be given in the form of a maſh, but if ſoap is preferred, it may be conveniently given in balls, moderately ſoftened. But among the claſs of ſolvents, the moſt active is the cauſtic mineral alkali, as will be ſhewn under the chemical analyſis, therefore, this ſeems the moſt eligible to

* See Whytt's Works, 4to, Edin. 1768, p. 447.

be made choice of. It may be incorporated with bran into a mafh, or with oil into a foap; and dilution fhould be employed to affift it *.

The two laft remedies I propofe to make mention of may rather be named antilithics

* There are two medicines of very great repute unnoticed in my clafs of lithontriptics, I mean, the aerial acid and the aerial mephitic alkali; the firft recommended by Drs. Hulme and Dobfon, the latter by Dr. Falconer—They are both medicines of great value, and highly deferving of notice; but as I have not been able to afcertain the lithontriptic powers of thefe remedies fufficiently to fpeak decidedly concerning them, particularly the latter, I have purpofely omitted the confideration of them. But for thofe who may wifh to make trial of the latter, or mephitic alkaline water, the following is the mode of preparing it: take an ounce, troy weight, of dry falt of tartar, put it into an open earthen veffel, pour on it fomewhat more than two quarts of the fofteft water that can be procured, and ftir them well together; after ftanding twenty-four hours, the clear part is carefully to be poured off, free from any indiffoluble refiduum that may remain, and put into the middle part of one of the glafs machines for impregnating water with fixed air, and expofed to a ftream of that fluid. After the water has remained in that fituation twenty-four hours, it will be fit for ufe, and fhould then be carefully bottled off into clear bottles, well corked, and fet with the bottom upwards, in a cool place. Half a pint of this has been taken three times a day in calculous cafes without difagreeing with the ftomach.

than than ſolvents; theſe remedies are the beards of leeks and uva urſi.

The beards of leeks have been a long while known among paupers, and ſeveral of them have informed me, upon enquiry, that they have experienced great relief from their uſe—ſome have aſſured me of their being cured by them, after having had the ſtone for years; and others have had their pains mitigated, and the ſtones evacuated with eaſe, when every thing elſe had been uſeleſs *. I have had ſome little experience of their powers in moderating the ſufferings of patients, by checking the ſeverity of paroxyſms, and leſſening the frequency of returns, therefore, am induced to aſſent to theſe aſſertions. A gentleman of veracity, and who had for years been afflicted with the ſtone, and was adviſed to the operation of lithotomy, aſſures me, that an infuſion of the beards of leeks, recommended to him by Mr. Cline, has afforded him the moſt eſſential relief †. It has enabled

* A handful of the beards of leeks may be infuſed in a pint of hot water, and the whole of this quantity taken daily.

† Two years have elapſed ſince this gentleman, Mr. Briſon, began to take the remedy, and the paroxyſms, which before enabled him to ufe exercife on horfeback, or in a carriage, without the fmalleft inconvenience; while previoufly to this, he was unable to ufe either, without bloody water, mucous difcharges, tenefmus, and other diftreffing fymptoms.

I have been lately informed, that the beards of leeks have been introduced into our hofpitals, and that many trials have been made with them, and much relief experienced from them; if fucceeding trials fhould confirm this, they will be a valuable medicine in lithiafis, whether inteftinal, veficular, or renal. In horfes I fhould imagine the dofe to be limited from two to four quarts of infufion; while a pint feems fufficient in the human fubject.

The next antilithic is uva urfi, on the action of which Dr. Cullen, who claffes it among the aftringents, has the following obfervations:

"In mentioning the general effects of aftrin-
"gents, I muft not omit their fingular power of
"relieving the fymptoms which attend the pre-
"fence of a calculus in the urinary paffages *."

before were frequent and fevere, are now long in their intervals, and too inconfiderable to be noticed.

* We may fafely infer their power when calculus is feated in other paffages.

" Among the differtations of De Heucher,
" formerly a Profeffor at Wittemburg, there
" is one under this title, *Calculus per ad-*
" *ftringentia pellendus*. In this he fhows,
" that almoft at all times, and by the moft
" eminent phyficians, aftringents have been
" employed in calculous cafes. He is, indeed,
" intent upon fhewing, that aftringents have
" been employed in promoting the excretion
" of calculi; but I prefume that, in the cafes
" in which thofe remedies appeared fuccefsful,
" the calculous matter was only fuppofed to be
" evacuated, becaufe the patient was relieved
" from the fymptoms that he formerly laboured
" under. But we now know that thefe fymp-
" toms may be relieved without the ftones
" having been diffolved or evacuated. A proof
" of this appears in the ufe of the leaves of
" the uva urfi, which not only from the expe-
" riments of the late Mr. De Haen, but alfo
" from my own, I have found to be
" often powerful in relieving the fymptoms of
" calculus. This plant is manifeftly a powerful
" aftringent; and in what manner this and
" other aftringents are ufeful in the cafes men-
" tioned, may be difficult to explain; but I
" fhall offer a conjecture on this fubject: I fup-

" pofe

" pofe their effect to depend upon their abforbing " acid in the ftomach. Their powerful attrac- " tion of acid we have mentioned above; and " that thereby they may be ufeful in calculous " cafes, is rendered probable by this, that the " medicines which of late have been found the " moft powerful in relieving the fymptoms of " calculus, are a variety of alkalines, which " are known to do this without their acting at " all in diffolving the ftone *."

Many authors, befides Dr. Cullen, relate inftances of relief in calculous cafes from medicines not lithontriptic; therefore, as they have no operation on the ftone, they probably operate on the membranes, by deftroying the fpecific irritation. Perhaps future inquiries may lead us to remedies fitted to prevent the predifpofition to concretion, as well as to deftroy the act of concretion when affumed. It has clearly been proved that this difeafe is the effect of irritation, of the mechanical kind, on membranes predifpofed to be affected; without this, mechanical irritation would always produce ftone, and every ftone have a nucleus; inftead of which, we find ftones can be generated with-

* Materia Medica, Vol. II. p. 12.

out out nuclei, and not generated when nuclei are prefent to produce them. Befides, fœtuffes have been found with ftones in their bladder *, and parents have tranfmitted the conftitutional predifpofition to their offspring.

I fhall here conclude the pathology, and mode of treatment of calculi in general, and of inteftinal calculi in particular; and fhall now confider their chemical properties, or the materials of which they are compofed.

### SECTION II.

### *Chemical Analyfis of the inteftinal Calculi of Horfes.*

The inteftinal calculi employed in thefe experiments were given to me by a perfon who obtains his livelihood by flaughtering horfes. The fpecimens I have procured from him may be divided into three claffes.

The firft clafs includes forty ftones, of various fizes and fhape, taken from the colon of a horfe. They have fmooth furfaces, and readily

* Doctor Monro and Mr. Cline both mention in their Lectures to have feen ftones in the urinary bladder of the fœtus; and Van Swieten relates the fame.

take take a poliſh; are of a dark-brown colour, and hard in their texture, but do not ſtrike fire with ſteel. The ſmalleſt weighs one ſcruple, the largeſt two ounces. In the ſmaller ſort the figure is pyramidal, in the larger, rhomboidal, with deep impreſſions on the ſurface. They are brittle, and ſplit by ſlight percuſſion, the fracture terminating with the external lamina; but if the ſtroke applied be conſiderable, the ſtones become cleaved in two, and diſcover in their centre a nucleus, which, in ſome, is an irregular pebble, in others a particle of iron*: round this the laminæ are accumulated, and their compoſition is beautifully regular, being ſtriated cryſtals curiouſly interwoven; their colour is brown and yellow.

Fig. 1, of Plate 1, repreſents one of the largeſt of theſe ſtones. A tranſverſe ſection of it ſhows the elegant arrangement of its cryſtals, in regular radii from the centre to the circumference. Its weight is an ounce and a half.

Fig. 2 (Plate 1) is an oval calculus, compoſed of numerous concentrated coats. A portion of it is broken off to ſhow this, and the

* The nucleus is not an invariable attendant, though obſervable in moſt of them.

To face Page 76.

Fig 6.

Fig 4.

Fig 1.

Fig 5.

Fig 7.

Fig 2.

Fig 3.

Josh. Cristal del. T. Ravenhill sculp.

line of demarkation difplays its thicknefs. Its weight is two ounces.

Fig. 3 (Plate I.) is a quadrangular ftone belonging to this clafs of fpecimens.

Figures 4 and 5 (Plate I.) fhow two fections of ftony fhells, broken from an entire oval ftone. The fmooth internal concave furface, and the radiated edge, with lines of feparation at right angles, difplay its concentrated ftructure and mode of cryftallization.

The fecond clafs includes but one ftone, fix pounds in weight, of a pale afh colour, rough on its furface, and found folitary in the great inteftine *. It is pyramidal in its form; hath three flat fides and three rounded edges; a flat bafe, and obtufe apex. It feems to be compofed of innumerable lamellæ, difpofed in circles from the centre to the circumference, without any difcernible nucleus. The denfity is different in different parts: about one inch from the furface it is folid and compact; the next inch and a half is fpongy; from this to the centre, it is as condenfed as marble. Vide Plate II.

* I am unacquainted with the age, feed, or peculiar fufferings of the animal from which this ftone was taken; but am informed that it excited inflammation, and proved fatal.

Fig.

Fig. 1 * ſhows its radiated and laminated appearance internally; Fig. 2 exhibits its external ſurface. At one angle ſeveral laminæ are removed to ſhow that the deep-ſeated coats are ſimilar to the more ſuperficial ones.

The third claſs includes two ſtones. The firſt of theſe (Fig. 3, Plate II.) is of a clay colour, and approaches in figure to an irregular ſphere. It is rough on its ſurface and friable in its texture; and is divided by a tranſverſe ſection to ſhow the irregularities of its internal ſtructure. Its weight is ten ounces. Of the ſecond calculus of this claſs two figures are given (ſee Figures 6 and 7 of Plate I.) This is alſo of a clay colour, is irregularly rounded, ſpongy in its texture, without lamellæ, and beſpangled with many black gliſtening cryſtals. Fig. 6 ſhows its nucleus, which proved to be a harneſs button. The weight of this calculus is ſeven ounces.

All theſe calculi, compared with diſtilled water, poſſeſs the ſame ſpecific gravity, being as 86 to 72.

* This and the other two figures of Plate II. are on a reduced ſcale of half their natural diameter: but all the figures of Plate I. are of their full ſize.

To face Page 78.

Fig 1.

Fig 2.

Fig 3.

Jos: Cristall del.

Ravenhill sculp.

### Experiment I.

### *Action of Heat on Inteſtinal Calculus.*

One drachm of inteſtinal calculus, calcined for the ſpace of four hours, in an open crucible, was reduced to a black gritty powder, and deprived of half its weight.

The ſame weight of calculus, with twice its weight of charcoal, calcined for the ſame ſpace of time, produced an aſh-coloured calx, not ſuſceptible of attraction to the magnet. When a few grains of ſalt of tartar were added to the mixture, or the black flux was employed in place of charcoal, no particles of metal were diſcoverable.

Twenty grains of calculus, in fine powder, being ſprinkled upon red hot iron, did not ſmoke, nor ſparkle, nor ſmell; but rolled upon the ſurface of the metal, and became changed to a dark brown granulated powder; half its weight being evaporated.

Ten grains of calculus, and one ſcruple of nitre, being triturated together in a mortar, were projected into a red hot crucible; a few red

ſparks were thrown up, and a ſlight hiſſing noiſe was heard, but no active deflagration was obſerved. What remained in the crucible was hard and tough, of a pale aſh colour, of little taſte, and difficult ſolubility in water.

By the action of heat ſo uniformly diſſipating one half of the weight of the calculus, and leaving the remainder a charred cinder, we may conclude ſome dry, oily, inflammable matter, and a large ſhare of ſublimable matter, to be reſident in theſe concretions: but nothing metallic like iron; for after calcination with charcoal, they diſplayed no attraction to the magnet.

### Experiment II.

*Action of Vitriolic Acid on Inteſtinal Calculus.*

Half an ounce of concentrated vitriolic acid by weight, being poured upon half a drachm of powdered calculus, excited no efferveſcence; but the acid loſt its colour and tranſparency, was changed to a deep black, and coagulated.

Six

Six drachms more of the acid being added, produced fluidity, and reduced the coagulum to the confiftence of coffee grounds. This being diluted with one ounce and a half of diftilled water, fome flight effervefcence arofe, and much heat was generated; the colour changed to a dark brown, and fluidity was greatly increafed; while the mixture feparated into two parts, one a light brown tranfparent liquid, the other a collection of dark brown flocculi, which floated upon the furface of the liquid.

In two days one half of thefe flocculi fubfided, but the remainder continued in fufpenfion. By agitating the phial, and then fuffering the mixture to fettle, the whole of the flocculi were precipitated. The fluid part was now feparated by filtration, and the flocculi collected on the filter.

Thefe flocculi were vifcid, of the confiftence and colour of thin treacle, of an acid tafte, empyreumatic fmell, and incapable of deficcation by heat. While expofed to heat they exhaled a moft fetid and fulphureous fmell, and the filter paper became fticky and glutinous.

Some of theſe flocculi, collected by filtration were lixiviated with diſtilled water, but no ſort of ſolution was effected. When alcohol was employed in place of water, one half of the flocculi were diſſolved, and conveyed through the pores of the filter. What remained unattacked by the alcohol was dark coloured, and capable of deſiccation, but adhered ſo firmly to the paper, that no ſeparation could be effected.

A filtered ſolution of calculus in vitriolic acid precipitated a pale brown powder, on ſaturating the acid with cauſtic vegetable alkali. This powder, when properly lixiviated and dried, amounted to three grains; and, when diſſolved in dilute vitriolic acid, taſted very ſtyptic and aluminous.

Another ſolution of calculus in concentrated vitriolic acid was diluted with two ounces of diſtilled water, the feculent matter ſeparated by filtration, and the ſolution evaporated; when ſome cryſtals were formed, ſmall, yellowiſh, and tranſparent, ſtyptic to the taſte, with a ſlight degree of bitterneſs. In attempting to evaporate to dryneſs, they melted into a dark-brown oil, very ſulphureous and offenſive.

Two ſcruples of calculus put into a glaſs retort, were expoſed to the heat of a ſpirit lamp, with one ounce weight of concentrated vitriolic acid, when the retort became filled with a white cloud, and the acid was converted into that which is volatile and ſulphureous.

If we attend to the phenomena preſented by theſe experiments, we may obſerve phlogiſton and argillaceous earth to be two of the conſtituent parts of calculus. The converſion of the acid into a black coagulum, and this, on the application of heat, being changed into volatile ſulphureous acid, demonſtrate the exiſtence of phlogiſton *; while the ſtyptic taſte of the dilute ſolution, and the aluminous ſalt formed by evaporation, demonſtrate the exiſtence of clay. The bitterneſs attending this ſalt may be attributed to the preſence of magneſia.

* In the preſent ſtate of chemiſtry I may be criticized, perhaps, for employing the term phlogiſton, or attempting to explain any of theſe phenomena by its agency; but as many chemical philoſophers in this country have not yet rejected the phlogiſton of Stahl for the pneumatic ſyſtem of Lavoiſier, I hope to be excuſed in the choice of it.

### Experiment III.

*Action of nitrous Acid on inteſtinal Calculus.*

Half an ounce of fuming nitrous acid being poured upon half a drachm of calculus, ſome ſlight effervefcence aroſe, and the acid was changed to a deep orange colour, with a copious diſcharge of red vapours. After the diſſipation of the vapour, the whole of the calculus was in ſolution, and the mixture became yellow and tranſparent. One ounce of diſtilled water being added, a violent effervefcence aroſe, and the colour became changed to green. When the effervefcence ceaſed, the yellow colour was aſſumed again. In a few days, ſome pale yellow flocculi were ſeparated, which were inconſiderable in quantity, and gradually ſubſided. The ſolution was paſſed through a filter, and then decompoſed by the cauſtic vegetable alkali, though every alkali was in poſſeſſion of this property. The precipitate was waſhed, and dried; when it amounted to twenty grains. If aërial alkali were employed, the powder acquired eight grains in weight; and if this again were were diſſolved in an acid, the eight grains were diſſipated in gas.

This precipitate was inſoluble in water, alcohol, or cauſtic volatile alkali; but was ſoluble in lime water, cauſtic vegetable alkali, and cauſtic mineral alkali; alſo in dilute vitriolic acid. With the alkalies it produced no change in their cauſticity, and a large quantity was neceſſary for ſolution. With the dilute vitriolic acid it produced a very ſtyptic and aluminous compound.

To a ſolution of calculus in nitrous acid ſome phlogiſticated alkali was added, to mark the preſence of iron, but no blue powder was precipitated; neither could calcareous earth be detected, either by the acid of ſugar, or concentrated acid of vitriol.

Forty grains of calculus were diſſolved in half an ounce of aqua fortis, this quantity of calculus being as much as the acid would take up. The ſolution, upon being evaporated in a glaſs ſaucer, cryſtallized into a lemon-coloured ſalt. This ſalt, by expoſure to the atmoſphere, attracted humidity, and one half melted into a pale yellow tranſparent liquid; while the other, in form of a whitiſh cake, was firmly adherent to the glaſs. The liquid part, amounting to two drachms, was decanted off, and tafted extremely bitter, like a ftrong infufion of orange peel. Two ounces of diftilled water being added to it, produced no alteration of its tranfparency; but *oleum tartari per deliquium* being dropped in, it inftantly became turbid, and a quantity of white powder was precipitated. This powder, which, after lixiviation and drying, amounted to fixteen grains, was as white as milk, and very fimilar to magnefia. Diluted vitriolic acid was poured upon this precipitate, when a flight effervefcence arofe, and three grains of gas were diffipated, which had probably been abforbed from the alkali. The folution was flowly effected, and though excefs of acid was employed, one grain refifted its action. After filtration and flow evaporation, fome tranfparent white cryftals were formed, very bitter, ftyptic, and acidulous.

As the nitrous acid, according to Bergman and Scheele, is capable of decompofing urinary calculi, and feparating an acid, *fui generis*, called the acid of calculus, in form of rofe-coloured cryftals, foluble in water, and capable ble of ftaining animal fubftances red; and as thefe celebrated chemifts have attributed the formation of calculus to the prefence of this acid in union with animal earth, I have beftowed peculiar attention, in my analyfis of inteftinal calculus, to look for the acid they defcribe. To difcover this, fome nitrous acid was faturated with inteftinal calculus, and though the folution was tranfparent, and of a pale yellow, yet, upon application to the fkin, no red coloured fpots were formed, which fhould have been effected, had the lithic acid been prefent: befides, the fkin was irrritated confiderably, fpotted yellow inftead of red, and incapable of ablution by water; while the rofe-coloured fpots defcribed by Scheele, were foluble in water, and no way irritating to the fkin.

Another portion of nitrated folution of inteftinal calculus was evaporated to drynefs, which, if the lithic acid were prefent, fhould have left a rofe coloured falt; but, in place of this, yellow-coloured cryftals were formed, one half of which was nitrated magnefia, the remainder an infipid white concrete, neither calcareous, aluminous, nor magnefian. The anonymous author, already quoted, in his new Theory of the Gout and of the Stone, relates, that the lithic acid is contained in the healthieſt urine, and is ſeparable from the ſame, in a cryſtalline form, by means of any other acid. To examine this precipitate, I collected ten grains, by adding a few drops of marine acid to eight ounces of recent urine, and frequently repeating the experiment. But after being collected, waſhed, and dried, inſtead of poſſeſſing the properties of an acid, it was inſoluble in water, inſipid to the taſte, and changed the blue infuſion of red-cabbage leaf, green; and inſtead of forming roſe-coloured cryſtals after ſolution and evaporation in nitrous acid, a yellowiſh white powder was left, which appeared to be animal earth. It preſented phenomena very ſimilar to the coagulable lymph of the blood; for it changed vitriolic acid black, and diſſolved; admitted of dilution with water to a certain extent, beyond which the acid was abſtracted, and moſt of the earth precipitated. The precipitate of urine was found ſoluble in the three mineral acids concentrated, and decompoſable by dilution with water; and coagulable lymph, ſimilarly treated, was found equally ſoluble in the concentrated acids, and equally decompoſable by water.

### Experiment IV.

*Action of Muriatic Acid on Intestinal Calculus.*

Half an ounce of concentrated muriatic acid being poured upon half a drachm of powdered calculus, the acid became dark brown, and little of the ſtone was taken up. This being diluted with one ounce meaſure of diſtilled water, ſome ſlight effervefcence aroſe; the colour changed to a light brown, and the mixture became tranſparent. In a few minutes it grew turbid, and many brown flakes were ſeparated and depoſited at the bottom of the phial. The ſolution being filtered, and then decompoſed by aërial vegetable alkali, eighteen grains of light brown precipitate were collected, which, when diſſolved in dilute vitriolic acid, taſted very ſtyptic and aluminous. This aluminous liquid was afterwards decompoſed by pure magneſia, when an aſh-coloured power ſubſided, which proved to be argillaceous.

In the experiments of Scheele on urinary calculus with muriatic acid, he found this acid incapable of producing ſolution, whether it be concentrated or diluted; but in my experiments with this acid on inteſtinal calculus, ten grains in thirty were taken up, whether the acid were concentrated or diluted, cold or expoſed to heat. Eighteen grains were ſpecified to have been precipitated, but eight of theſe were the aërial acid abſorbed from the alkali employed.

### Experiment V.

*Action of Acetous Acid on Inteſtinal Calculus.*

Six drachms of diſtilled vinegar, digeſted with thirty grains of calculus, became dark brown, and turbid. When diſtilled water was added to the mixture, a ſlight efferveſcence took place, the colour became paler, with quick ſeparation of ſediment. The mixture being filtered, was decompoſed by the vegetable alkali, when a light brown powder was precipitated, amounting to fourteen grains. This, diſſolved diffolved in weak vitriolic acid, was ftyptic, bitter, and aluminous.

One fcruple of calculus was digefted four-and-twenty hours with half an ounce of diftilled vinegar. This was filtered, and the clear liquor evaporated, when fix grains of a pale brown falt were collected, without any fenfible tafte, but completely foluble in two-ounce meafures of diftilled water.

### Experiment VI.

*Action of Aërial Acid on Inteftinal Calculus.*

Ten grains of each fpecimen of calculus, digefted three days in ten ounces of diftilled water impregnated with fixed air, were completely diffolved by the menftruum, excepting one grain.

### Experiment VII.

*Action of Aërial Vegetable Alkali on Inteftinal Calculus.*

Twenty grains of calculus were triturated in a mortar with twenty grains of falt of tartar, and and four ounces of diſtilled water. The mixture was filtered through blotting paper, and the reſiduum, when dried, amounted to fifteen grains. Theſe fifteen grains being triturated with the ſame weight of ſalt of tartar, and four freſh ounces of diſtilled water, only two grains paſſed through the filter. By repeating this experiment ſeven times more, with the ſame weight of alkali as of ſtone, and four ounces of water with each trituration, the whole of the calculus was in ſolution; ſo that thirty-ſix ounces of water with 101 grains of Alkali, were requiſite for the ſolution of twenty grains of calculus, aided by repeated triturations.

Though aërial vegetable alkali, aided by trituration, rendered calculus ſoluble in water, yet a ſaturated ſolution of the ſame alkali in water had no ſolvent power, when the trituration was omitted. This is ſimilar to what happens in the action of magneſia on Peruvian bark, (remarked by my late worthy and ingenious friend, Dr. Skeete *) for magneſia, whether calcined

* Experiments and Obſervations on Quilled and Red Peruvian Bark. 8vo. London, 1786, p. 54.

or or uncalcined, promoted the folution of the active parts of bark in water, if affifted by trituration, but fhewed no fort of folvent influence on bark by fimple maceration in a phial.

When calculus was triturated with dry alkali, and a few drops of water were fuperadded, a volatile alkaline odour was evolved; but if quick lime were added inftead of vegetable alkali, in the proportion of four fcruples of lime to two fcruples of ftone, and heat applied to the retort, fome cauftic volatile alkali was collected.

Volatile alkali is a falt abundant in animal matter, and moftly neutralized by the acid of phofphorus; and according to the experiments of Scheele, Bergman, Higgins, and Auftin, on human calculus, it is found to be part of its compound; therefore, inteftinal calculus of the quadruped being an animal production, the prefence of this falt might be expected.

### Experiment VIII.

*Action of Cauftic Vegetable Alkali on Inteftinal Calculus.*

Forty grains of folid calculus infufed in fix ounces of cauftic vegetable alkali, imparted a deep deep-brown colour to the lixivium, and loſt twenty-five grains of its weight; but no complete ſolution could be effected. If the alkaline lixivium were diluted with five times its weight of water, the external ſurface became bleached, without one grain diminution in weight.

### Experiment IX.

*Action of Cauſtic Mineral Alkali on Inteſtinal Calculus.*

A ſmall calculus, of a brown colour, and weighing thirty-three grains, was digeſted three days, in two ounces of cauſtic mineral alkali; when the external ſurface became pale, was reduced into white flocculi, and ſeparated by the ſlighteſt agitation. The calculus being removed from the lixivium, well waſhed in diſtilled water, and dried, was found ſeven grains diminiſhed in weight: the external lamina was in ſolution, the next ſeparated in flakes, and the third loſt its colour and texture. The remaining twenty-ſix grains were returned into the phial, and digeſted two days with one ounce of freſh alkali; when the ſurface of the calculus, which which was deeply corroded, ſeparated from the interior lamina, leaving a dark brown central body, beſtudded with numerous ſpiculæ of white, tranſparent, and oblong cryſtals. Theſe cryſtals, mildly ſaline in their taſte, were a pure *ſal ſodæ*, which only could have been formed by the attraction of the alkali to ſome mephitic acid contained in the ſubſtance of the ſtone, as the air of the atmoſphere was excluded. By repeated applications of freſh cauſtic alkali, the reſidue of the calculus was diſſolved, excepting three grains, which no quantity of alkali would act on.

As the cauſtic mineral alkali ſeemed a powerful ſolvent of calculus, and promiſed to be a good lithontriptic, I attempted to aſcertain its power in dilution. For this purpoſe, ſeven grains of ſolid calculus were infuſed twenty-four hours in one ounce meaſure of this alkali and four ounce meaſures of diſtilled water, when the calculus loſt one grain in weight: by the ſecond application one grain more; by the third two grains and an half; by the fourth half a grain; by the fifth one grain; and one grain remained unaffected.

### Experiment X.

*Action of Caustic Volatile Alkali on Intestinal Calculus.*

Twenty grains of solid calculus, macerated in two ounces of caustic volatile alkali, were not in the smallest degree lessened in weight, nor changed in colour or texture. The same quantity of calculus in fine powder, being macerated together for three days, was two grains diminished in weight, but no farther solution could be effected.

### Experiment XI.

*Action of Lime Water on Intestinal Calculus.*

Eight grains of dark-brown calculus, infused in eight ounce measures of recent lime water, lost nothing of their weight or colour; but eight grains of ash-coloured calculus, infused in eight ounce measures of lime water, were three grains lessened in weight. The grey coloured calculus calculus loſt two grains in weight, but no farther ſolution could be effected.

From theſe experiments with alkaline ſalts and lime water, we may obſerve the mineral and vegetable alkalies to be powerful ſolvents, while cauſtic volatile alkali and lime water are conſiderably inferior in their powers.

The ſolubility of inteſtinal calculus by alkaline ſalts ſhows its great affinity to the urinary calculus of the human body, analyzed by Scheele, Bergman, Lane, Higgins, and Auſtin. All theſe chemical philoſophers found the human calculus ſoluble in alkaline menſtrua and lime water; but this ſolubility was not uniform; for it differed in different calculi, and in different laminæ of the ſame calculus. But one thing may be worthy of notice, that the mineral alkali was always the moſt powerful agent, and the facility of ſolution was always in proportion to their volatility by fire.

Some calculi are with difficulty ſoluble in alkaline lixivia; for Dr. Black had a calculus from the human bladder, of an ounce weight, which took two years to complete its ſolution; and he has ſeen patients, under an alkaline medicinal courſe which had no obvious ſolvent effects.

### Experiments XII. XIII.

*Action of Water on Inteſtinal Calculus.*

Firſt, *Cold Water*—Half a drachm of calculus in powder, infuſed three days in two ounce meaſures of diſtilled water, and agitated, ſhewed no diſpoſition for ſolution. After agitation it ſeparated into two diſtinct parts, one a fine light brown powder, capable of being ten minutes ſuſpended; the other a dark brown heavy powder, which quickly precipitated to the bottom. The liquid part with the light coloured powder in ſuſpenſion, was decanted from the droſs, and then filtered, dried, and carefully weighed, when it amounted to twelve grains: while the heavy brown powder, ſimilarly examined, weighed eighteen grains; which added to the other made thirty grains, being the amount of the quantity infuſed.

Eight grains of calculus being infuſed in eight ounces of cold water for three days, one grain appeared in ſolution; but no additional quantity of of water ſeemed capable of diſſolving the remainder.

Secondly, *Boiling Water*—Twelve grains of powdered calculus boiled in a florentine flaſk with twelve ounces of diſtilled water, diſſolved two grains, which paſſed through the pores of the filter; but no farther ſolution could be effected.

The watery ſolution was divided into five diſtinct portions, and cauſed the following metallic precipitations; extract of lead white; vitriol of iron light grey; vitriol of zinc milk white; vitriol of copper green: and when mixed with lime water, the latter immediately became turbid, without any ſenſible diminution of cauſticity.

From the action of fire and acids upon theſe ſpecimens of inteſtinal calculus, they appear to be compoſed of the following ſubſtances, varioully proportioned and combined, viz. dry animal oil—animal gelatinous matter—volatile alkali—argillaceous earth and magneſia. I alſo ſuſpected phoſphorus acid to be preſent from the reſult of the following experiment:

Some powdered calculus, charcoal duſt, and ſalt of tartar, were put into a two-ounce phial. This was placed in a large crucible, and the ſpaces filled up between the phial and ſides of the crucible with ſand. After two hours expoſure to a cinder fire in a common bath ſtove, the ſides of the phial in contact with the ingredients, and the ingredients themſelves, became one ſolid vitrefied maſs.

My ſuſpicion, however, with reſpect to the exiſtence of phoſphoric acid in theſe concretions, has ſince been proved to be not well founded, by the following experiment, made at Guy's Hoſpital, by my ingenious friend Mr. Babington.

Six ounces of dark brown calculus, in powder, were put into a florentine flaſk, with four ounces of concentrated vitriolic acid, and twice that weight of diſtilled water—The mixture was expoſed to heat, and gently boiled for about thirty minutes, after which it was immediately filtered. The flaſk was now waſhed with eight freſh ounces of diſtilled water, which being gently heated, were thrown into the filter with the former. The clear ſolution, amounting to ſomewhat more than a pint, was unavoidably left for about two days, when it was decanted for the purpoſe of being evapoevaporated; and the bottle was found incrufted with a collection of moft beautiful cryftals.—Thefe cryftals, perfectly tranfparent, were white in colour, and varioufly fhaped—eafily foluble, and when put upon the tongue tafted mildly faline and bitter. Their upper furface was thickly befet with needle-fhaped fpiculæ, or white, tranfparent, and hair-like cryftals, which were quite infipid, difficultly foluble in water, and decompofable by acid of fugar. To prove the bafis of the larger cryftals, a few grains were triturated with quick-lime, when a pungent odour was evolved, which proved to be volatile alkali. Therefore volatile alkali, and vitriolic acid formed the compound of the larger cryftals; while calcareous earth, and the fame acid, formed the compofition of the fpiculæ. But as Epfom falt was fufpected to be prefent in the larger cryftals, from the bitternefs of their tafte, fome cryftals were diffolved in water, and then decompofed by vegetable alkali, when a milk white powder was dropped, which re-diffolved in vitriolic acid, and proved to be pure magnefia.

The filtered liquor of vitriolic folution, after decantation from the cryftals, was evaporated to the confiftence of a fyrup, then mixed with charcoal duſt, and diſtilled in a retort; but though much volatile ſulphureous acid, and hepar of ſulphur came over, nothing like phoſphorus was diſcernible. Therefore a large ſhare of phlogiſton, volatile alkali, and magneſia, were diſcoverable in this ſtone, but only a very minute portion of lime, and no ſenſible quantity of phoſphorus.

## Figures and Tables

**Fig 1. Fig 2. Fig 3. Fig 4. Fig 5. Fig 6. Fig 7. f1:**
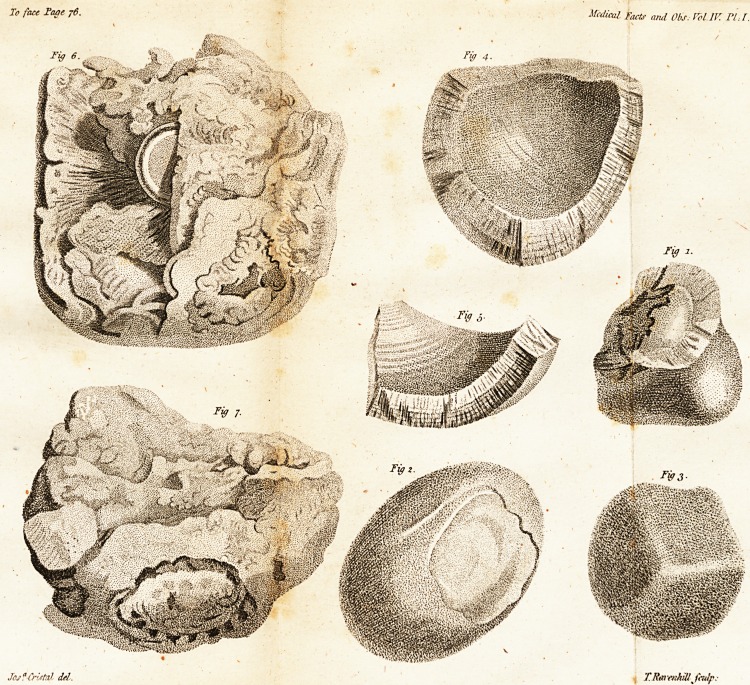


**Fig 1. Fig 2. Fig 3. f2:**